# Evaluating a Telehealth Coaching and Mobile-Based Digital Engagement Intervention for People With Cancer Using the Patient-Reported Outcomes Measurement Information System Global Health: Pilot Questionnaire Study

**DOI:** 10.2196/72647

**Published:** 2026-04-01

**Authors:** Joanne Lewis, Shen Wang, Toni Rose Jue, Tim Atkins, Scott A Irwin, Puneet Nanda, Davinder Sangar, Lynette Mackenzie, Raghav Murali-Ganesh

**Affiliations:** 1 Osara Health Eveleigh, NSW Australia; 2 Faculty of Medicine, Nursing, Midwifery and Health Sciences The University of Notre Dame Sydney Australia; 3 Cedars-Sinai Cancer, and Department of Psychiatry and Behavioral Neurosciences Cedars-Sinai Health System Los Angeles, CA United States; 4 University of Bristol Bristol United Kingdom; 5 Faculty of Medicine and Health University of Sydney Sydney Australia

**Keywords:** cancer, digital, telehealth, coaching, mental health, physical health

## Abstract

**Background:**

People with cancer often experience unmet needs during treatment and survivorship, which can impact their ability to carry out daily tasks, reduce their quality of life, and limit their participation in work and social activities. Cancer Coach by CancerAid (now known as Osara Health) is a digital health intervention designed to address these needs through a combination of synchronous telehealth coaching and an asynchronous mobile app that supports behavior change and emotional well-being.

**Objective:**

This study aimed to evaluate the impact of Cancer Coach by CancerAid on the mental and physical health of people with cancer using patient-reported outcomes.

**Methods:**

Participants were referred to the program via insurers and hospital clinics. Health coaches administered the Patient-Reported Outcomes Measurement Information System 10-item Global Health Short Form via telephone at both the beginning and end of the intervention. This tool measures global physical health (GPH) and global mental health (GMH). Pre- and postintervention scores were analyzed using Wilcoxon signed rank tests. Independent 2-tailed *t* tests assessed whether changes in GPH and GMH scores were associated with the use of health coaching alone or in combination with the mobile app.

**Results:**

Statistically significant improvements were observed in both GPH (*z*=−4.97; *P*<.001; *r*=0.37) and GMH (z=−4.53; *P*<.001; *r*=0.34), indicating moderate effect sizes in the 89 participants. The average T score point changes of 4.43 for GPH and 4.58 for GMH represented a minimal important change for participants. The improvement in the group GMH T score was reflected in the move from “good” to “very good” mental health status. Participants who engaged with both health coaching and the mobile app showed greater improvements in physical health, whereas those who received health coaching alone exhibited higher gains in mental health. This suggests that the mode of support may influence specific health outcomes.

**Conclusions:**

The use of the Patient-Reported Outcomes Measurement Information System 10-item Global Health Short Form showed that participants had significant improvements in physical and mental health after participating in the Cancer Coach by CancerAid️ intervention. The integration of telehealth coaching with app-based support may enhance overall well-being and address holistic needs during cancer treatment and survivorship.

## Introduction

Countries with strong health care systems have seen increased cancer survival rates due to improvements in early detection and treatment of cancer [1]. However, cancer treatment side effects can impact physical, cognitive, psychological, and emotional well-being, not only in the acute phase of treatment but also for months or years afterward [2]. Managing the immediate and long-term side effects can be further complicated by health care funding shortages and a lack of services [3]. Worldwide, rehabilitation options for addressing the long-term holistic survivorship needs of people with cancer, including resuming everyday roles and occupations and improving quality of life, are limited [4]. Health coaching and various digital health options have emerged as a way of addressing some of the unmet needs of people with cancer [5].

Health coaching is a collaborative and client-centered intervention that aims to improve the physiological, behavioral, psychological, and social well-being of people with chronic illnesses [6]. For people with cancer, it offers a holistic approach to managing overall health and well-being beyond that of the primary oncology care team [7], assisting with informational, behavioral, and social concerns, particularly after treatment. A longitudinal study of 3-month telephone health coaching with survivors of cancer showed significant improvements in patient-reported depression, anxiety, exercise, and quality of life. The improvements continued for 6 months after coaching ceased, with slight declines at 12 months [8]. Participants indicated that health coaching assisted with improving exercise and healthier eating, with the most helpful aspect being the motivation and feedback provided by a personal health coach [8]. Health coaching for survivors of cancer was also supported by a systematic review [9], which found improvements in quality of life, mood, and physical activity.

Health coaches use various techniques, including motivational interviewing, goal setting, and behavior change strategies, to support and empower people to improve their overall health [10]. Technological advances have increased digital and telehealth options and reduced the need for traditional in-person health coaching, particularly since the global COVID-19 pandemic as a way of staying connected with people during periods of isolation [11]. Digital and telehealth options are also beneficial to those in rural areas with community access difficulties or caring or work responsibilities [12]. Purcell et al [13] used the Patient-Reported Outcomes Measurement Information System 10-item Global Health Short Form (PROMIS-10) to assess changes in veterans’ health status following participation in a telehealth coaching program. The study found significant improvements in PROMIS-10 physical and mental health scores after coaching, indicating that the telehealth coaching program positively influenced veterans’ overall health and well-being [13].

For people with cancer, digital and telehealth support options can help alleviate the financial burdens associated with attending in-person appointments [14] and provide valuable assistance during treatment periods when they may be immunocompromised. Mobile health (mHealth) options in cancer care can encompass activation and support of self-management, exercise, and enablement of survivorship care delivery [15]. Specifically, mHealth applications vary in use from treatment side effect and symptom management, virtual medical consultations, exercise prescription, support groups, and education and care navigation [16]. The method of delivery can be synchronous (eg, telephone calls or virtual care) or asynchronous (eg, wearable devices, web-based platforms, mobile-based apps, mobile-based digital games, and SMS text messages) [16,17]. Digital health programs can improve global, physical, and mental health while providing support in an ambulatory setting for people with cancer in managing their disease symptoms and side effects [16] by enhancing patients’ self-efficacy, as well as helping health care professionals track and respond to patient-reported outcomes [7]. Furthermore, digital cancer health coaching programs have also been found to improve physical and mental health scores [5,18,19]. However, most studies focus on a single mHealth intervention, and there is a paucity of research evaluating interventions that combine synchronous and asynchronous mHealth interventions.

Cancer Coach by CancerAid integrates synchronous coaching with asynchronous mobile support guided by the transtheoretical model [20]. The transtheoretical model conceptualizes behavior change as a progression through stages, from precontemplation to maintenance. This framework informed the structure of the intervention by aligning synchronous coaching with the earlier stages of change, incorporating motivational interviewing and goal setting to enhance readiness and intention. The asynchronous mobile app was designed to support participants across all stages but, in particular, in the action and maintenance stages through reminders, self-monitoring tools, and educational content that reinforces ongoing behavior change [20].

Previously, Cancer Coach by CancerAid has been found to improve people’s ability to return to work compared with age- and disease-matched individuals who did not participate in the program [21]. The primary aim of this pilot study was to determine whether Cancer Coach by CancerAid (now known as Osara Health) improved participants’ self-reported physical and mental health using PROMIS-10 scores [22-27], and a secondary aim was to explore which programmatic aspect contributed to these changes. This was to inform program improvements and modifications for an international cohort.

## Methods

### Study Recruitment

People with cancer were informed about the Cancer Coach by CancerAid program by their insurer, employer, or health service and self-enrolled into the program. CancerAid supports people at any time during their cancer experience. CancerAid coaches contacted people who self-enrolled and advised them about the program and the study.

Participants were matched using email addresses and mobile phone numbers to prevent multiple identities for users. Data were collected from August 2020 to November 2022 using secure online forms and databases. This period was chosen due to the consistency of having the same 3 health coaches. The end point was set to analyze data on broader app changes to enhance app integration into synchronous health coaching and ensure its relevance and applicability across an international cohort.

Eligibility criteria included a cancer diagnosis, completion of the PROMIS-10 survey before and after the program, and participation in at least 2 health coaching calls. Health coaches administered the PROMIS-10 questionnaire via phone before the first and after the final coaching call using a standard script for consistency (Multimedia Appendix 1). Use of the CancerAid app (Multimedia Appendix 2) was optional.

### Study Design

This study used a nonexperimental evaluative research design to assess the effectiveness of the overall intervention and the programmatic elements that contributed to its effectiveness [28]. All participants in the study received health coaching and access to the CancerAid app. During the study period, there were minor app bug fixes, content changes, and downtimes to enhance performance and security. Details about this can be found in Multimedia Appendix 3. These interruptions did not significantly affect the participants during the study period. As no new features were introduced and app maintenance changes were back end in nature, these were not expected to materially affect user experience or outcomes. Therefore, they were not controlled for in the statistical analysis.

### PROMIS-10 Measure

The PROMIS-10 is a standardized and validated tool [29] that established the methodological rigor for this study. The PROMIS-10 contains 10 questions related to the patient’s physical, mental, and social health and well-being. The complete PROMIS-10 questionnaire can be found in Multimedia Appendix 1. Patients rate their well-being on a 5-point Likert scale. One question related to pain is rated on a scale from 1 to 10. This score for pain is recomputed to a score out of 5. All 10 question scores are then recalculated to present 2 domain scores: global physical health (GPH) and global mental health (GMH). Both GPH and GMH have a maximum possible score of 20 points. For all items, a higher score equals more of the concept being measured, and a lower score indicates less of the concept being measured. Multimedia Appendix 1 also provides inclusions for calculating GPH and GMH scores.

Although the PROMIS-10 is primarily used to generate composite scores for GPH and GMH, this study also analyzed individual item responses to explore specific symptom changes. While not standard practice, item-level analysis can yield clinically relevant insights [30], particularly in cancer populations where symptoms such as pain, fatigue, and emotional distress are common. These insights may help refine health coaching strategies and enhance understanding of how coaching influences distinct aspects of functioning.

The GPH and GMH scores were recalibrated using the same underlying metric, which corresponds to a normalized T score. The T scores are standardized to the general US population. The average T score for the US population is 50 points, with an SD of 10 points [26,29,31]. PROMIS-10 domains use different cutoff score ranges. The PROMIS-10 GPH score ranges are as follows: excellent (T score>58), very good (T score=50-57), good (T score=42-59), fair (T score=41-35), and poor (T score<35). The PROMIS-10 GMH score ranges are as follows: excellent (T score>57), very good (T score=48-56), good (T score=40-47), fair (T score=39-29), and poor (T score<29) [26].

To identify minimal important change [32] or “meaningful change” [26] in PROMIS-10 scores, a review of the literature found that a 2- to 6-point change in T scores for PROMIS-10 measures could be reasonably considered as an indicator [32]. Minimal important change has been defined as a threshold for a minimal within-person change over a period during which patients perceive themselves to have experienced an important change [32].

### Intervention

Cancer Coach by CancerAid is a flexible, evidence-based program designed to support people with cancer over approximately 6 to 12 weeks. It combines telehealth coaching with a mobile app and resources to address key areas such as symptom tracking, nutrition, sleep, exercise, psychosocial well-being, and return to work. The educational content is based on American Society of Clinical Oncology guidelines and findings from large randomized trials, ensuring that topics such as lifestyle interventions and digital symptom tracking are backed by the latest evidence. Support is delivered via a combination of asynchronous digital resources (app, SMS text messages, and emails) and synchronous coaching calls, of which there are approximately 3 throughout the program.

Each participant is paired with a qualified health coach (with backgrounds in allied health, nursing, or medicine) trained in standardized methods that ensure the scope of practice while allowing for a client-centered approach. Coaches provide tailored interventions based on the individual’s diagnosis, stage of cancer, and specific needs. They use evidence-based behavior change techniques, including goal setting and review, symptom monitoring, motivational interviewing, feedback and accountability, positive reinforcement, lifestyle education, and social and emotional support. The approach is based on the transtheoretical model of change, with an emphasis on empowering participants to take proactive steps toward improving their health and well-being. Regular communication and digital support help overcome barriers to face-to-face care, creating a supportive framework for sustained self-management and behavior change.

Further information about the Cancer Coach by CancerAid program can be found in the study by Lo et al [21].

Health coaching is complemented by the CancerAid app (now known as the Osara Health app), an mHealth app providing symptom tracking, health information articles, and supportive care resources. Key features include symptom logs, evidence-based articles, and a note-taking feature (Multimedia Appendix 2). The app was designed using principles of behavioral science and patient-centered care, incorporating elements such as self-monitoring and educational empowerment. The development process of the CancerAid iOS and Android app followed the typical iterative agile development process of planning, design, development, testing, deployment, and review. The app was tested through an automated test suite in addition to manual testing by developers and stakeholders. Throughout the study period, no new features were introduced, and the new releases were limited to maintenance updates. The app was relatively stable during the study period and is archived in a private code repository owned by CancerAid. The app is free and publicly accessible via the Apple App Store and Google Play Store. Participants were encouraged by health coaches to use the app to supplement their health coaching calls.

### Statistical Analysis

Physical and mental health were evaluated using the PROMIS-10 measure to determine the impact of Cancer Coach by CancerAid. An examination of the data distribution revealed no evidence of a ceiling effect; scores were well distributed across the full range, indicating adequate variability for analysis. Descriptive statistics were used to establish ranges and means of variables. Tests for normality showed that the data did not fit normal distributions, and therefore, nonparametric testing was used. Raw PROMIS-10 GPH and GMH scores were converted to T scores to compare with US population norms [26,29,31]. A T score of 50 (–10 to +10) is considered to be a healthy score, and well-being descriptors are ascribed based on scores and associated categories (see PROMIS-10 Measure section) [26,33]. Two-tailed *t* tests were used to determine the correlation between the number of calls and app use and changes in GPH and GMH scores. For an exploratory study on individual item score changes, the Wilcoxon signed rank test was used to compare the paired groups to determine whether there was a statistically significant difference (*P*≤.05). The effect size of the Wilcoxon signed rank test was calculated and considered in terms of the classification of effect sizes by Cohen [34] (0.1=small effect; 0.3=moderate effect; ≥0.5=large effect) [29,34]. Data were analyzed using SPSS (version 29; IBM Corp).

### Ethical Considerations

Ethics approval was obtained from the University of Sydney Human Research Ethics Committee (approval 2024/HE000297). Informed consent was obtained from all individual participants to take part in the research. Participation in the study was voluntary. Participant privacy and confidentiality were protected throughout the study in accordance with institutional, national, and international guidelines for human subjects research. No compensation was provided. All data were deidentified prior to analysis, and no identifying information is included in the published results.

## Results

### Participants

Between August 2020 and November 2022, a total of 200 participants completed a PROMIS-10 survey at either the start or end of the intervention, 93 (46.5%) of whom had both pre- and posttest data. A total of 2% (4/200) of the participants were excluded due to missing data. The 55% (107/200) attrition rate was due to participants not completing the postprogram PROMIS-10 survey for health, family, or work reasons, resulting in a final dataset of 89 participants. Table 1 provides demographic data.

Analysis between the group included in the study (completed pre- and posttest PROMIS-10 questionnaires; n=89) and those not included (did not complete the posttest PROMIS-10 questionnaire; n=111) was conducted. An independent-sample 2-tailed *t* test compared the mean ages of the 2 groups, revealing no significant difference (mean age 51.44 years; *P=*.35). Cross-tabulation analyses of gender showed no significant association (33/111, 30% male; *P*=.73). However, the chi-square test indicated a significant difference between the groups on diagnosis (*P<*.001). Notably, the nonstudy group had 4 times as many cases of an unknown cancer diagnosis compared to the study group. The top 5 cancer types in the group not included in the study were breast, unknown, bowel, blood, and lung. Employers comprised a larger proportion of the referral sources in the nonstudy group (employer: 11/111, 10%; insurer: 92/111, 83%; hospital: 8/111, 7%).

**Table 1:** Participant demographics (N=89).

Characteristic		Values
**Sex, n (%)**		
	Male	32 (35.9)
	Female	57 (64)
	Intersex	0 (0)
Age (years), mean (SD; range)		52.86 (9.94; 23-82)
**Cancer type, n (%)**		
	Breast	34 (38.2)
	Blood	14 (15.7)
	Bowel	8 (9)
	Head and neck	8 (9)
	Prostate	5 (5.6)
	Brain	4 (4.5)
	Gynecological	4 (4.5)
	Melanoma	4 (4.5)
	Unknown	4 (4.5)
	Kidney	2 (2.2)
	Other (single cases: pancreas, liver, lung, spinal cord, and neuroendocrine)	5 (5.6)
**Referral source, n (%)**		
	Insurer	78 (87.6)
	Hospital	10 (11.2)
	Employer	1 (1.1)
Duration of program (days), mean (SD)		52 (32.5)

### PROMIS-10 Global Health Scores and T Scores

A statistically significant mean change of 4.43 points was observed in the GPH T score (*P<*.001); however, the score remained within the “Good” category of well-being. Similarly, the mean GMH T score increased by 4.48 points following participation in the Cancer Coach by CancerAid intervention, a change that was also statistically significant (*P<*.001). The mean postintervention GMH T score was 49.052 (SD 7.4992), indicating an improvement in overall mental health status from “Good” to “Very good.” Both the 4.43-point change in the GPH T score and the 4.48-point change in the GMH T score are considered clinically significant. Table 2 outlines changes in both GPH and GMH T scores.

**Table 2:** Global physical health and global mental health T scores.

		Before the intervention	After the intervention	Mean change (points)		*P* value	
**Global physical health**					4.43		.001
	T score, mean (SD)	42 (6.745)	47.09 (6.655)				
	Category	Good	Good				
**Global mental health**					4.58		.001
	T score, mean (SD)	44.47 (8.233)	49.052 (7.4992)				
	Category	Good	Very good				

Pearson correlations revealed no significant relationship between age and changes in GPH score (*r*=0.003; *P*=.98) or GMH score (*r*=−0.178; *P*=.10). Independent-sample 2-tailed *t* tests showed no significant gender differences in GPH score change for male (mean 2.16, SD 3.08) and female (mean 1.60, SD 2.93) individuals (*P*=.40). For GMH score change, means were 4.27 (SD 9.32) for male individuals and 4.76 (SD 8.10) for female individuals (*P*=.80). One-way ANOVA showed significant differences in both GPH score change (*P*=.009) and GMH score change (*P=*.001) across diagnostic groups. Post hoc analyses (Tukey honestly significant difference) indicated that participants with head and neck cancers had substantially higher GPH and GMH score changes compared with those in several other groups. The combined model including all 3 predictors (age, sex, and diagnosis) was assessed using multiple linear regression analyses. Neither the model for GPH score change (*F*_3, 85_=0.30; *P=*.83; *R*^2^=0.01) or that for GMH score change (*F*_3, 85_=1.16; *P*=.33; *R*^2^=0.04) were significant. None of the individual predictors contributed significantly to either model.

### Number of Health Coaching Calls and Changes in GPH and GMH Scores

**Figure 1:**
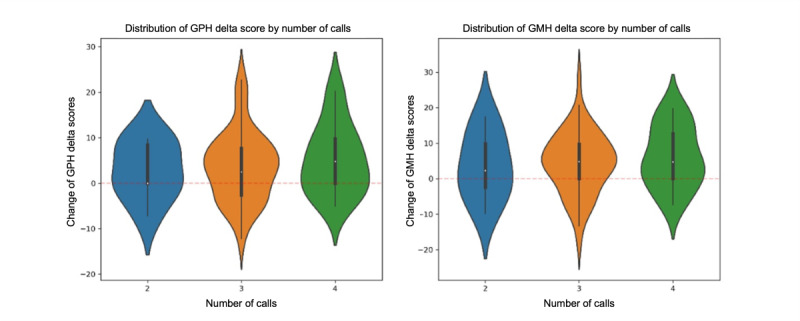
Distribution of global physical health (GPH) and global mental health (GMH) scores according to number of health coaching calls.

For the call frequency and app usage analysis, 6 of the 89 participants were excluded because they completed more than 4 calls. These values fell outside the expected intervention range and were considered outliers in program engagement. The final analytic sample for this section, therefore, included 83 participants. Figure 1 illustrates the distribution of changes in physical health (GPH) and mental health (GMH) scores across different numbers of health coaching calls. While statistical analysis using ANOVA (Tukey honestly significant difference) showed no significant differences between call frequencies (GPH: *P<*.69; GMH: *P<*.74), the consistently wide range of positive score changes for 3, 4, and 5 calls suggests that patients can experience meaningful improvements regardless of call frequency. The presence of positive median values (shown by the white dots in Figure 1) for most call groups indicates an overall trend toward improvement in both physical and mental health scores, with slightly more improvement in physical and mental health with more calls.

### Use of the App and Changes in GPH and GMH Scores

**Figure 2:**
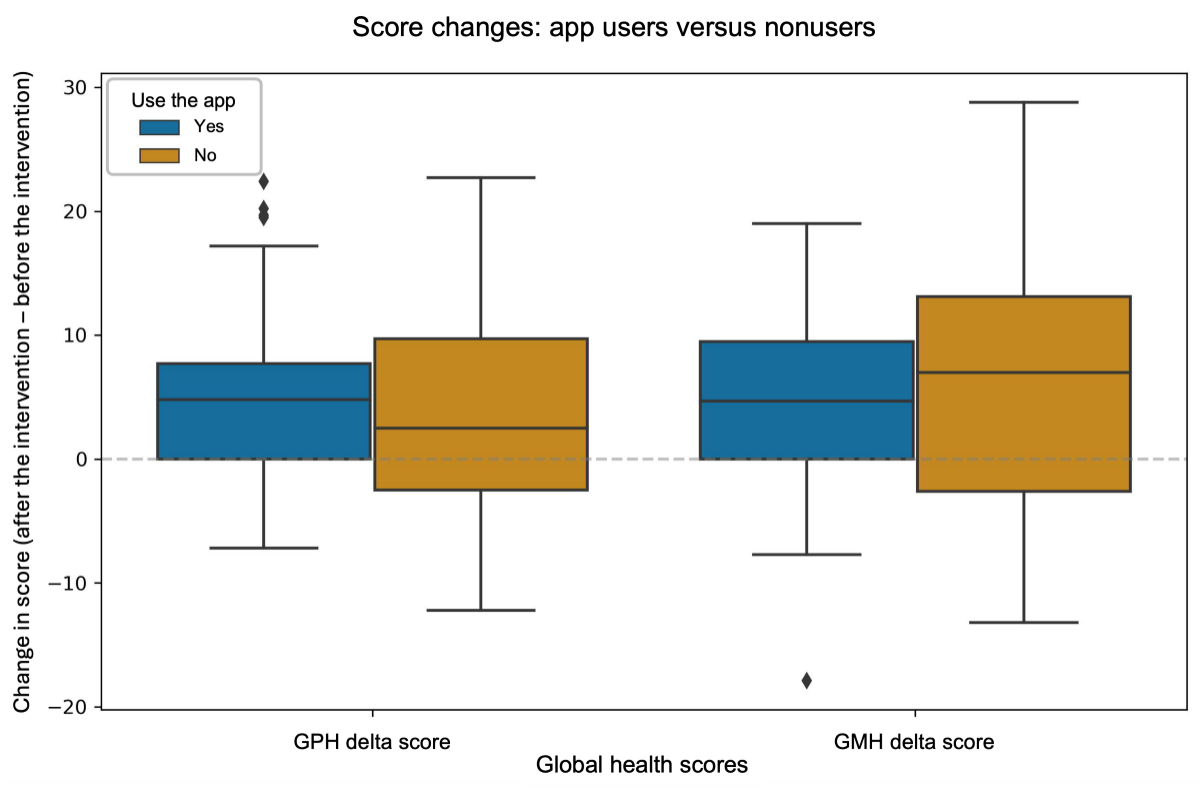
Median and distribution of global physical health (GPH) and global mental health (GMH) scores for health coaching with app use and only use and health coaching calls with no app use.

Table 3 outlines the change in GPH and GMH scores between app users (53/83, 64%) and nonusers (30/83, 36%) in the Cancer Coach by CancerAid intervention. Figure 2 shows that both groups exhibited overall positive improvements in their health scores, with median values notably above 0, indicating general health benefits regardless of app use. Non–app users (health coaching calls only) showed slightly higher median improvements in GMH scores 4.74 (SD 10.61) compared to app users (with health coaching calls) 4.63 (SD 7.29); whereas app users (with health coaching calls) had a higher median GPH score change 4.29 (SD 7.15) than participants who only had health coaching calls 3.22 (SD 8.07).

**Table 3:** Global physical health (GPH) and global mental health (GMH) scores for app users and non–app users.

	GPH point score change	GMH point score change
App users (median)	4.29 (SD 7.15)	4.63 (SD 7.29)
Non–app users (median)	3.22 (SD 8.07)	4.74 (SD 10.61)
*t* test (*df*)	0.62 (81)	–0.05 (81)
*P* value	.53	.96

## Discussion

### Principal Findings

This study aimed to evaluate the effectiveness of the Cancer Coach by CancerAid program using patient-reported outcomes and how these outcomes were related to the quantity of health coaching calls and mobile app use.

A significant number of participants had a higher GPH score upon completion of the Cancer Coach by CancerAid intervention, and the improvement was deemed clinically significant. GPH includes items of general physical health, ability to carry out daily activities, pain, and fatigue. Many mHealth programs aim to improve physical health with a focus on exercise oncology [35]. However, participants in the Cancer Coach by CancerAid program improved their physical well-being through a more holistic approach to mHealth via support for management of pain and fatigue levels and addressing a range of barriers to participation in everyday activities, including work [21,36]. Studies have shown mixed results in terms of mHealth programs reducing fatigue and pain levels [17,37,38]. The significant change in GPH score in this study could be related to improvements in fatigue and pain levels (see Multimedia Appendix 4 for individual score results in this study). Participating in the Cancer Coach by CancerAid program appears to be related to improved physical health through a personalized goal setting and psychoeducation approach rather than an exercise oncology focus. These findings contribute to the growing body of research supporting the benefits of nonpharmacological interventions to improve physical well-being and fatigue experienced by people with cancer. The magnitude of improvement observed in PROMIS-10 scores in this study is consistent with findings of other digital health coaching interventions. For example, there have been similar gains in PROMIS-10 GPH and GMH scores reported among veterans receiving telephone-based coaching [13]. Chow et al [5] also found improved mental health using a mobile coaching intervention among survivors of cancer. These improvements in PROMIS-10 scores, particularly in domains such as fatigue, emotional well-being, and social roles, have important implications for participants’ broader cancer experiences [5]. They may reflect enhanced self-efficacy, functional recovery, and psychosocial adjustment across the different stages of diagnosis, treatment, and survivorship.

A significant number of participants had a higher GMH score upon completion of the Cancer Coach by CancerAid intervention, and the improvement was considered clinically significant. GMH includes items of quality of life, mental health (mood and cognition), social activities and relationships, and emotional well-being (anxiety and depression). Importantly, the average change in GMH T score moved the overall score of the group from a rating of “good” to a rating of “very good” mental health at the end of the program. Although not a counseling service, there are psychosocial aspects of the Cancer Coach by CancerAid intervention that support emotional well-being and improve participants’ sense of agency, which was possibly related to the improved mental health scores. These results are similar to those of recent systematic reviews that have shown that mHealth interventions effectively reduce anxiety and depression in patients with cancer [39] and improve quality of life [16].

Cancer Coach by CancerAid provides holistic care, addressing the physical and emotional support needs specific to the participant regardless of whether they are early or later in their cancer experience. The improved GPH and GMH scores indicate that the program could be providing this. Similarly, a holistic approach, such as health coaching, can also be beneficial in addressing aspects of health and well-being in a collective way [9,40]. A previous study of Cancer Coach by CancerAid also indicated that participation assisted people with cancer in resuming work activities [21]. This holistic approach could also provide a more effective form of health service delivery as it targets a wider group of people with cancer. If a digital and telehealth coaching program can assist with improving pain, fatigue, and mental and physical health, this may also have a cost benefit by reducing health care use, such as general practitioner consultations [41] and hospital visits [42,43].

There was inconclusive evidence as to which mHealth programmatic aspect of the Cancer Coach by CancerAid program contributed to the positive GPH and GMH scores. Participants who only had health coaching calls and no app use had slightly higher GMH scores than those who used both the app and the calls. However, participants who used the app and health coaching calls had a slightly higher GPH score than participants who only had health coaching calls. It is possible that the synchronous telephone health coaching aspect of Cancer Coach by CancerAid had more influence on participants’ mental health and well-being than the asynchronous app, but this requires further investigation. Overall, the comparable outcomes between app users and nonusers suggest that patients can benefit from the Cancer Coach by CancerAid program through multiple engagement pathways, allowing for flexibility in how they choose to participate in their care journey. Additionally, the flexibility in call frequency may be beneficial for personalizing support schedules to individual patient needs and circumstances rather than requiring a fixed number of calls for all patients, acknowledging that higher GPH and GMH score changes were experienced by those with more calls, although this was not significant. These findings are similar to those of a systematic review that examined synchronous, asynchronous, and mixed approaches to delivering mHealth interventions to patients with cancer and found that the mode of delivery did not influence outcomes [17]. Understanding the most effective form of delivery is critical for developing evidence-based practice and cost-effective service delivery, so 3-arm randomized controlled trials are recommended [17] to better understand the effectiveness of mHealth programmatic aspects and health outcomes.

### Limitations

Pilot studies are often conducted to obtain information in preparation for a larger trial. Therefore, the findings of this study should be interpreted with an understanding of the relatively small sample size and the absence of a control group. While all participants during this study completed the Cancer Coach by CancerAid program, the 55% (107/200) attrition rate refers specifically to noncompletion of the postprogram PROMIS-10 survey. This lack of follow-up data may have biased the study results toward those who experienced greater improvement as those who did not complete the survey may have had less positive outcomes. Therefore, it is possible that a higher survey completion rate could have revealed weaker overall effects.

Because Cancer Coach by CancerAid supports participants at any stage of the cancer journey, certain stages may result in better or worse scores. Although analysis showed no significant differences in age or gender between participants included in the study and those excluded due to missing postintervention PROMIS-10 data, there were notable differences in cancer diagnosis and referral source. A significantly higher proportion of excluded participants had an unknown cancer diagnosis, and the distribution of cancer types varied, potentially impacting the representativeness of the study sample. Additionally, a greater proportion of excluded participants were referred by employers rather than insurers or hospitals, suggesting possible differences in case characteristics or motivations for program enrollment. These factors may limit the generalizability of the findings to all program participants.

While there may be a lack of information about the stage of cancer and participants’ details, this is seen as a benefit for our participants, who can be cautious about oversharing their personal and health information. This approach may also increase the number of people willing to enroll in the Cancer Coach by CancerAid program. The participants were 64% (57/89) female, with breast cancer being the dominant diagnosis, which may contribute to gender bias but also reflects men’s reluctance to seek psychological or emotional help when dealing with cancer [44]. Additionally, race and ethnicity data were not collected as part of this study. This aligns with CancerAid’s model of minimizing data collection to reduce barriers to participation but limits the ability to evaluate cultural or demographic variability in outcomes.

Health coaching is provided to meet individual needs and goals and uses techniques of goal setting and motivational interviewing, among others. All coaches were trained using the same models and methodologies to ensure consistency across techniques used and program reliability. Although the coaches followed consistent methodologies, differences in communication style and participant responsiveness could lead to variability in the results. To reduce potential bias with health coaches administering the PROMIS-10 survey, alternative approaches should be considered. A self-administered digital option within the app could offer greater privacy and autonomy, although it may risk lower completion rates. Another option is to have the survey administered by a neutral third party, which could help preserve objectivity while maintaining engagement.

Further studies on this program will need to focus on more robust data collection on app engagement, comparison with a control group, and extended use of the PROMIS-10 to evaluate sustained changes in functioning. Qualitative studies should consider content analysis of coaching calls to ascertain techniques affecting change and focus groups to gain participants’ personal views and experiences of the Cancer Coach by CancerAid program. Given the multiple options in the delivery of mHealth solutions and the variance in reporting interventions in research, more evidence is required to establish the effectiveness of specific modes of delivery as well.

### Conclusions

Participants with various cancer diagnoses showed a significant and positive change in physical and mental health after taking part in the Cancer Coach by CancerAid program as indicated through the PROMIS-10 survey. These preliminary results suggest that clinical practice, including a mixed delivery of synchronous (telehealth coaching) and asynchronous (mobile app) interventions, provides holistic care and may improve physical and mental health and well-being for those with cancer. This type of intervention appears to constitute a potential option for patients with cancer who have unmet needs within traditional health care systems.

## Appendix

Multimedia Appendix 1 Ten-item Patient-Reported Outcomes Measurement Information System questionnaire.

Multimedia Appendix 2 Screenshots of the CancerAid (now known as Osara Health) app.

Multimedia Appendix 3 CancerAid iOS release notes.

Multimedia Appendix 4 Individual PROMIS-10 question score results.

## Abbreviations

GMH: global mental health
GPH: global physical health
mHealth: mobile health
PROMIS-10: Patient-Reported Outcomes Measurement Information System 10-item Global Health Short Form
