# Supplementary material for: Evaluating a Telehealth Coaching and Mobile-Based Digital Engagement Intervention for People With Cancer Using the Patient-Reported Outcomes Measurement Information System Global Health: Pilot Questionnaire Study
Source: JMIR Cancer. 2026 Apr 1;12:e72647. doi: 10.2196/72647 (PMC13085991; doi:10.2196/72647)
Supplement: Multimedia Appendix 1 [file cancer_v12i1e72647_app1.docx]

Appendix 1: PROMIS 10 Questionnaire

Instructions:

1. Preamble

Osara Health is always looking to improve its Coach Program. I'm wondering if you would mind participating in a short 10 question survey today and again at the end of the program? It will help us understand if and how the program supports you and where we can look to improve'

1. Score Options

| Q1-6: For the following 6 questions, you can provide an answer from 1 to 5 (1=poor, 2=fair, 3=good, 4=very good and 5=excellent) | | | | | |
| --- | --- | --- | --- | --- | --- |
| Question | 5 | 4 | 3 | 2 | 1 |
| Q1.In general, would you say your health is? | Excellent  ᆦ | Very Good  ᆦ | Good  ᆦ | Fair  ᆦ | Poor  ᆦ |
| Q2.In general, would you say your quality of life is? | Excellent  ᆦ | Very Good  ᆦ | Good  ᆦ | Fair  ᆦ | Poor  ᆦ |
| Q3. In general, how would you rate your physical health? | Excellent  ᆦ | Very Good  ᆦ | Good  ᆦ | Fair  ᆦ | Poor  ᆦ |
| Q4. In general, how would you rate your mental health, including your mood and your ability to think? | Excellent  ᆦ | Very Good  ᆦ | Good  ᆦ | Fair  ᆦ | Poor  ᆦ |
| Q5. In general, how would you rate your satisfaction with your social activities and relationships? | Excellent  ᆦ | Very Good  ᆦ | Good  ᆦ | Fair  ᆦ | Poor  ᆦ |
| Q6. In general, please rate how well you carry out your usual social activities and roles?  (This includes activities at home, at work and in your community, and responsibilities as a parent, child, spouse, employee, friend, etc.) | Excellent  ᆦ | Very Good  ᆦ | Good  ᆦ | Fair  ᆦ | Poor  ᆦ |
| Q7: For the following question, you can provide an answer from 1 to 5 (1=not at all, 2=a little, 3=moderately, 4=mostly and 5=completely) | | | | | |
| Question | 5 | 4 | 3 | 2 | 1 |
| Q7. To what extent are you able to carry out your everyday physical activities such as walking, climbing stairs, carrying groceries, or moving a chair? | Completely  ᆦ | Mostly  ᆦ | Moderately  ᆦ | A little  ᆦ | Not at all  ᆦ |
| Q8: For the following question, you can provide an answer from 1 to 5 (1=always, 2=often, 3=sometimes, 4=rarely and 5=never) | | | | | |
| Question | 5 | 4 | 3 | 2 | 1 |
| Q8. In the past 7 days, how often have you been bothered by emotional problems such as feeling anxious, depressed or irritable? | Never  ᆦ | Rarely  ᆦ | Sometimes  ᆦ | Often  ᆦ | Always  ᆦ |
| Q9: For the following question, you can provide an answer from 1 to 5 (1=very severe, 2=severe, 3=moderate, 4=mild and 5=none) | | | | | |
| Question | 5 | 4 | 3 | 2 | 1 |
| Q9. In the past 7 days, how would you rate your fatigue on average? | None  ᆦ | Mild  ᆦ | Moderate  ᆦ | Severe  ᆦ | Very Severe  ᆦ |
| Q10: For the following question, you can provide an answer from 0 to 10 (10 = worse pain imaginable and 0 = no pain) | | | | | |
| Question | 0 1 2 3 4 5 6 7 8 9 10 | | | | |
| Q10. In the past 7 days, how would you rate your pain on average? | ᆦ ᆦ ᆦ ᆦ ᆦ ᆦ ᆦ ᆦ ᆦ ᆦ ᆦ  no pain worse pain  imaginable | | | | |

1. Calculating Scores

Step 1: Recalculate Q10: Pain from score out of 10 to score out of 5:

| Q10 Score | 0 | 1 | 2 | 3 | 4 | 5 | 6 | 7 | 8 | 9 | 10 |
| --- | --- | --- | --- | --- | --- | --- | --- | --- | --- | --- | --- |
| Recalculated Score | 5 | 4 | 4 | 4 | 3 | 3 | 3 | 2 | 2 | 2 | 1 |
| New Q10 Score |  | | | | | | | | | | |

Step 2: Calculating Global Physical Health Raw Score: Add the scores as per table below:

| Question | Q3. In general, how would you rate your physical health? | Q7. To what extent are you able to carry out your everyday physical activities? | Q9. In the past 7 days, how would you rate your fatigue on average? | Q10. In the past 7 days, how would you rate your pain on average? (recalculated from above) | Global Physical Health Raw Score |
| --- | --- | --- | --- | --- | --- |
| Score | + + + = | | | | |

Step 3: Calculating Global Mental Health Raw Score: Add the scores as per table below:

| Question | Q2.In general, would you say your quality of life is? | Q4. In general, how would you rate your mental health, including your mood and your ability to think? | Q5. In general, how would you rate your satisfaction with your social activities and relationships? | Q8. In the past 7 days, how often have you been bothered by emotional problems such as feeling anxious, depressed or irritable? | Global Mental Health Raw Score |
| --- | --- | --- | --- | --- | --- |
| Score | + + + = | | | | |
