# Supplementary figures and images for: Evaluating a Telehealth Coaching and Mobile-Based Digital Engagement Intervention for People With Cancer Using the Patient-Reported Outcomes Measurement Information System Global Health: Pilot Questionnaire Study
Source: JMIR Cancer. 2026 Apr 1;12:e72647. doi: 10.2196/72647 (PMC13085991; doi:10.2196/72647)

Appendix 2: Screenshots of CancerAid (now known as Osara Health™) app


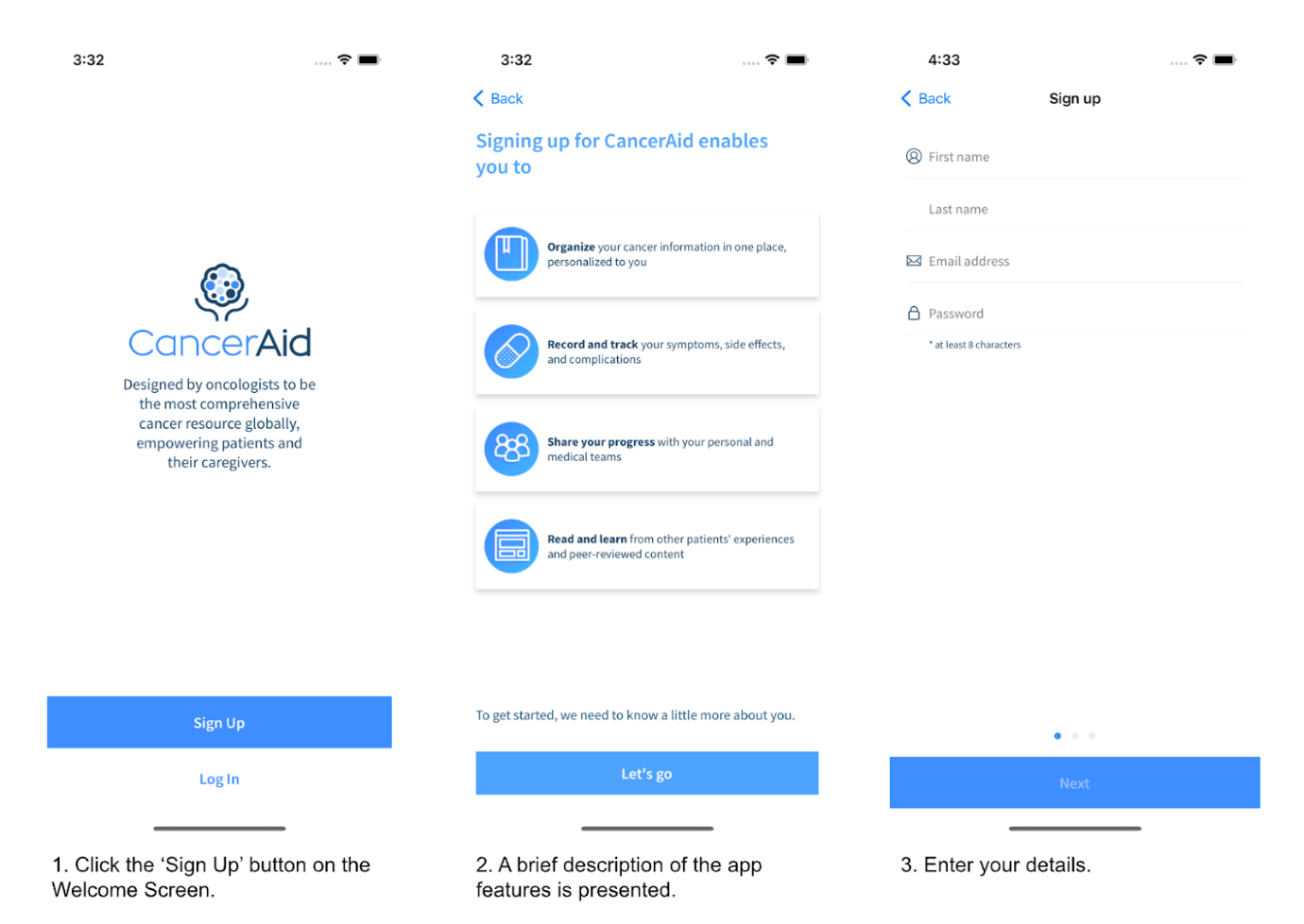

Supplement: Multimedia Appendix 2 [file cancer_v12i1e72647_app2.docx]

Appendix 3: CancerAid iOS Release Notes


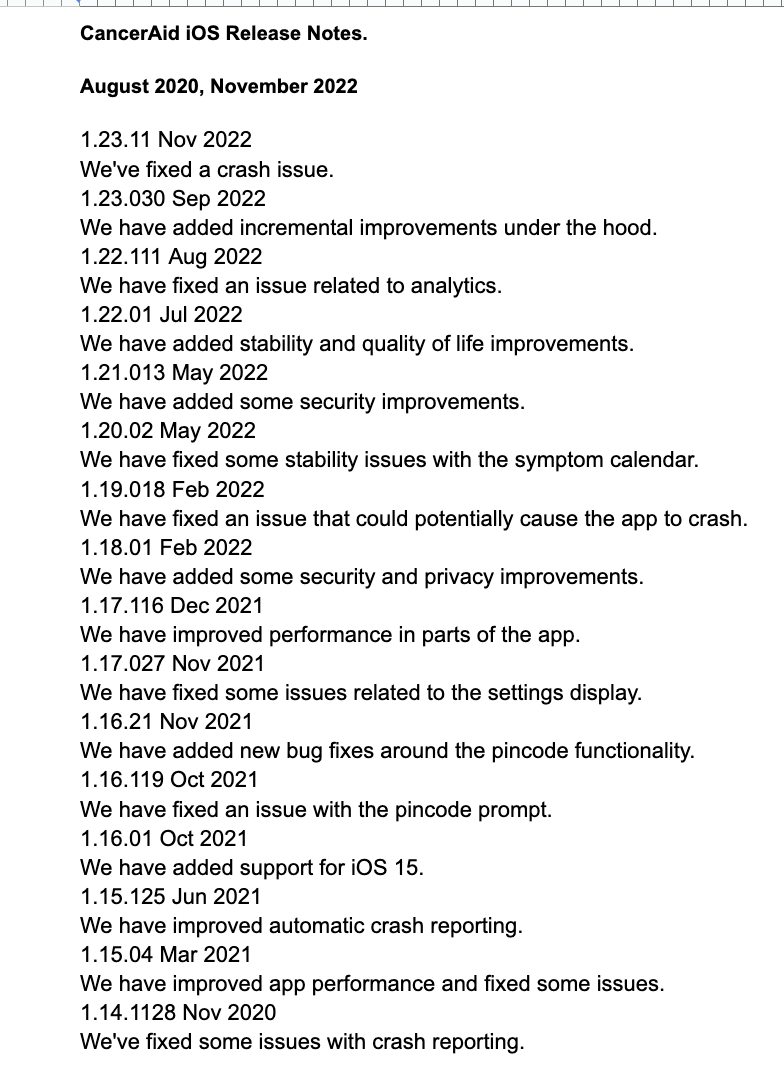

Supplement: Multimedia Appendix 3 [file cancer_v12i1e72647_app3.docx]
