# Supplementary material for: Evaluating a Telehealth Coaching and Mobile-Based Digital Engagement Intervention for People With Cancer Using the Patient-Reported Outcomes Measurement Information System Global Health: Pilot Questionnaire Study
Source: JMIR Cancer. 2026 Apr 1;12:e72647. doi: 10.2196/72647 (PMC13085991; doi:10.2196/72647)
Supplement: Multimedia Appendix 4 [file cancer_v12i1e72647_app4.docx]

**Supplementary file**

Although not the primary focus of this study, exploring changes in individual PROMIS-10 item scores offered meaningful insights into the lived experiences of participants with cancer. These item-level shifts provided health coaches with valuable feedback on which aspects of the program were resonating most, such as improvements in fatigue, emotional well-being, or social support and highlighted areas that may require more targeted attention during coaching calls. Additionally, this granular feedback can inform future enhancements to app design, ensuring that digital health tools are responsive to the nuanced and evolving needs of people navigating cancer care.

As shown in Table 1, all individual PROMIS-10 items, except Q10 Pain, had significant (*P<*.05) positive changes in pre and post scores and effect size. The largest changes were in Q7 Everyday physical activities (z=-4.181; *P<*.001), and moderate effect size of r=0.31, Q9 Fatigue (z=-3.900; *P<*.001 and small effect size of 0.29, Q3. Physical Health (z=-4.202; *P<*.001) and a moderate effect size of r=0.31Q8 Emotional Wellbeing (z=-3.411; *P<*.001) and a small effect size of 0.26 and Q1 General Health (z=-4.118; *P<*.001) and moderate effect size of r=0.31. There was a significant positive change in GPH, z=-4.966, *P=*<.001, with more than a moderate effect size of r=0.37 following participation in Cancer Coach by Osara Health^TM^. There was also a positive change in GMH, z=-4.533, *P<*.01, with more than a moderate effect size of r=0.34 after the intervention.

Table 1: Pre- and post- PROMIS 10

|  | | | | Wilcoxon Signed Ranked Test for Effect Size | | |
| --- | --- | --- | --- | --- | --- | --- |
| Question | Pre Mean | Post Mean | Mean  Change (points) | z value | *P*  (two tailed) | Effect Size (r) |
| Q1. General Health | 2.76 | 3.21 | 0.45 | -4.118 | <.001 | 0.31 |
| Q2. QOL | 3.18 | 3.57 | 0.39 | -3.144 | .002 | 0.24 |
| Q3. Physical Health | 2.53 | 3.01 | 0.48 | -4.202 | <.001 | 0.31 |
| Q4. Mental Health | 3.0 | 3.43 | 0.43 | -3.442 | <.001 | 0.26 |
| Q5. Social Activities | 3.18 | 3.65 | 0.47 | -3.393 | <.001 | 0.25 |
| Q6. Usual social roles and activities | 2.7 | 3.13 | 0.43 | -3.265 | .001 | 0.24 |
| Q7. Everyday physical activities | 2.98 | 3.57 | 0.59 | -4.181 | <.001 | 0.31 |
| Q8. Emotional wellbeing | 3.01 | 3.49 | 0.48 | -3.411 | <.001 | 0.26 |
| Q9. Fatigue | 2.63 | 3.13 | 0.5 | -3.900 | <.001 | 0.29 |
| Q10. Pain | 3.51 | 3.72 | 0.21 | -1.665 | .096 | 0.12 |
| Global Physical Health | 11.64 | 13.44 | 1.8 | -4.966 | <.001 | 0.37 |
| Global Mental Health | 12.37 | 14.15 | 1.78 | -4.533 | .001 | 0.34 |

Further changes in self-reported physical and mental health were evaluated using the Wilcoxon Signed Rank Test (Table 2). Positive Ranks indicate the number of participants with PROMIS-10 scores that were higher after the Cancer Coach by Osara Health^TM^ intervention. The highest mean positive ranks were Q9 Fatigue (34.98), Q8 Emotional Health (32.18) and Q4 Mental Health (30.91).

Table 2: Pre- and post- PROMIS 10 Wilcoxon Signed Ranked Test Mean Results

|  | | | |
| --- | --- | --- | --- |
|  | | **N** | **Mean Rank** |
| Q1. General Health | Negative Ranks | 13 | 25.12 |
|  | Positive Ranks | 43 | 29.52 |
|  | Ties | 33 |  |
|  | Total | 89 |  |
| Q2. Quality of Life | Negative Ranks | 17 | 27.85 |
|  | Positive Ranks | 41 | 30.18 |
|  | Ties | 31 |  |
|  | Total | 89 |  |
| Q3. Physical Health | Negative Ranks | 12 | 23.63 |
|  | Positive Ranks | 42 | 28.61 |
|  | Ties | 35 |  |
|  | Total | 89 |  |
| Q4 Mental Health | Negative Ranks | 16 | 29.38 |
|  | Positive Ranks | 44 | 30.91 |
|  | Ties | 29 |  |
|  | Total | 89 |  |
| Q5 Social Activities | Negative Ranks | 16 | 26.00 |
|  | Positive Ranks | 41 | 30.17 |
|  | Ties | 32 |  |
|  | Total | 89 |  |
| Q6 Usual social roles and activities | Negative Ranks | 16 | 28.38 |
|  | Positive Ranks | 42 | 29.93 |
|  | Ties | 31 |  |
|  | Total | 89 |  |
| Q7 Everyday physical activities | Negative Ranks | 13 | 25.88 |
|  | Positive Ranks | 45 | 30.54 |
|  | Ties | 31 |  |
|  | Total | 89 |  |
| Q8 Emotional Wellbeing | Negative Ranks | 17 | 29.71 |
|  | Positive Ranks | 45 | 32.18 |
|  | Ties | 27 |  |
|  | Total | 89 |  |
| Q9 Fatigue | Negative Ranks | 18 | 27.83 |
|  | Positive Ranks | 47 | 34.98 |
|  | Ties | 24 |  |
|  | Total | 89 |  |
| Q10 Pain | Negative Ranks | 19 | 27.00 |
|  | Positive Ranks | 33 | 26.21 |
|  | Ties | 37 |  |
|  | Total | 89 |  |
| Global Physical Health | Negative Ranks | 23 | 27.52 |
|  | Positive Ranks | 59 | 46.95 |
|  | Ties | 7 |  |
|  | Total | 89 |  |
| Global Mental Health | Negative Ranks | 23 | 32.54 |
|  | Positive Ranks | 60 | 45.63 |
|  | Ties | 6 |  |
|  | Total | 89 |  |
